# Characteristics of Flue Gas Dechlorination by Ethanol-Digested Calcium Oxide and Its Effect on Mercury Speciation and Concentration

**DOI:** 10.3390/ma19173588

**Published:** 2026-08-24

**Authors:** Shuzhou Wei, Yongzheng Gu, Jianshan Li, Chengzhe Shen, Xintong Wen, Hailong Liu, Tao Yang, Yunxia Shao, Xiaoshuo Liu

**Affiliations:** 1 GD Power Development Co., Ltd., Beijing 100025, China; 2 Beijing Guodian Electric Power Co., Ltd., Beijing 100101, China; 3 National Energy Coal-Based CCUS Technology R&D Center, Beijing 100101, China; 4 CHN Sanhe Power Generation Co., Ltd., Sanhe 065201, China; 5 Guoneng (Shandong) Energy and Environment Co., Ltd., Jinan 250013, China; 6 School of Information and Electronic Engineering, Shandong Technology and Business University, Yantai 264005, China; liuxiaoshuo@sdtbu.edu.cn

**Keywords:** flue gas dechlorination, competitive reaction, injection dechlorination, density functional theory, mercury speciation and concentration

## Abstract

This study aims to investigate the feasibility of ethanol-digested calcium oxide (CaO-E) as a novel dechlorination sorbent for the efficient removal of hydrogen chloride (HCl) from coal-fired flue gas and further evaluate its influence on mercury speciation and transformation in flue gas, thereby addressing the low efficiency and limited multi-pollutant control capability of conventional dry dechlorination technologies. Based on a laboratory-scale injection reaction system, ethanol-digested calcium-based sorbents were injected into simulated coal-fired flue gas to systematically examine the effects of key factors, including Ca/Cl molar ratio, SO_2_, and fly ash, on dechlorination efficiency. Density functional theory (DFT) calculations were further employed to elucidate the reaction mechanisms. Meanwhile, mercury-laden flue gas was introduced to investigate the removal characteristics of elemental mercury (Hg^0^) and oxidized mercury (Hg^2+^) by CaO-E. The experimental results demonstrated that ethanol-digested CaO exhibited significantly superior performance compared with untreated samples, and the formation of a porous calcium hydroxide structure was identified as the key factor responsible for its high dechlorination efficiency. When the Ca/Cl molar ratio reached 4.0, the dechlorination efficiency could be stably maintained above 80%. SO_2_ showed a pronounced inhibitory effect on the dechlorination process, whereas fly ash exhibited a slight promoting effect. Mercury removal experiments revealed that CaO-E had limited removal capability toward Hg^0^ but effectively reduced the concentration of Hg^2+^. Specifically, when the Ca/Cl molar ratios were 3 and 5, the Hg^2+^ concentrations decreased to 1.4 and 0.6 μg/m^3^, respectively. This behavior can be attributed to the fact that Hg^2+^ mainly exists in chlorinated forms such as HgCl_2_, which possess strong polarity and can be readily adsorbed by the alkaline active sites on the CaO-E surface. In addition, as the dechlorination process proceeded, chlorine-containing species in the flue gas were gradually consumed, suppressing the oxidation conversion of Hg^0^ to Hg^2+^ and thereby further reducing the Hg^2+^ concentration. Theoretical calculations indicated that both HCl and SO_2_ could undergo chemisorption on calcium active sites, while HCl possessed a lower reaction energy barrier and therefore dominated the competitive adsorption process, exhibiting preferential reactivity. Overall, ethanol-digested calcium oxide not only demonstrates excellent HCl removal performance, but also shows the capability to regulate mercury speciation in flue gas to a certain extent, providing both theoretical insights and technical support for the synergistic control of multiple pollutants in coal-fired flue gas.

## 1. Introduction

In recent years, the large-scale co-combustion of high-chlorine coal in Xinjiang (e.g., the Shaerhu coalfield), as well as biomass, coal sludge, and anti-freezing agents, has increasingly exacerbated chlorine-related emissions in coal-fired power plant flue gases [1,2,3,4,5,6]. Chlorine species in flue gas not only accelerate high-temperature corrosion of heat-exchange surfaces, shortening boiler lifetime, but also lead to the enrichment of chloride in by-products such as gypsum and wastewater, increasing the difficulty of subsequent resource utilization and wastewater treatment [7]. Meanwhile, the chlorine element also affects mercury (Hg) transformation and migration in flue gas by promoting the oxidation of elemental mercury (Hg^0^) to more easily removable divalent mercury (Hg^2+^), thereby influencing existing multipollutant control strategies [8,9,10]. Therefore, achieving efficient removal of chlorine species from flue gas is of critical importance for reducing equipment corrosion, mitigating secondary pollution, and enhancing multipollutant control efficiency.

From a policy perspective, in September 2024, the Ministry of Ecology and Environment of China issued the Emission Standard for Co-Processing of Solid Waste in Coal-Fired Boilers (Draft for Comment), which proposes limiting HCl emissions in flue gas to 10 mg/Nm^3^ [11]. As emission standards become increasingly stringent, the development of economically viable and high-efficiency flue gas dechlorination technologies suitable for industrial applications has become a pressing research priority. To address chlorine pollution in coal-fired flue gas, the U.S. Environmental Protection Agency (EPA) proposed a flue gas upstream dechlorination strategy to directly remove HCl, thereby reducing subsequent equipment corrosion and chloride-related risks [12,13]. Follow-up studies by research institutions such as Tsinghua University and Southeast University indicate that the key limitation of upstream flue gas dechlorination lies in the low reactivity of conventional dechlorinating agents (e.g., calcium hydroxide and sodium bicarbonate), which results in insufficient dechlorination efficiency for practical coal-fired power plant applications [14,15,16].

Recent studies have shown that high-activity calcium-based dechlorinating agents prepared via ethanol digestion can significantly enhance the pore structure and specific surface area of calcium hydroxide, increasing the number of reactive surface sites and thereby strengthening HCl capture [17,18,19]. In addition, the alkaline active sites formed by high-activity calcium materials may also affect mercury (Hg) in flue gas, which is one of the most hazardous heavy metal elements in coal-fired flue gas. Mercury in flue gas exists in two principal forms: elemental mercury (Hg^0^) and divalent mercury (Hg^2+^), predominantly as HgCl_2_ [8]. These two species exhibit markedly different physicochemical properties. Hg^0^ is chemically inert and relatively difficult to remove, whereas Hg^2+^ is water-soluble and tends to be captured by fly ash or enriched in wet flue gas desulfurization (WFGD) slurries [20]. Given this distinction, it is essential to systematically evaluate how flue gas dechlorination affects the concentrations and speciation behavior of both mercury forms.

On one hand, it may facilitate the adsorption of divalent mercury species such as HgCl_2_; on the other hand, by modifying the local chemical environment, they influence the oxidation behavior of Hg^0^, thereby affecting mercury speciation and its transformation and migration characteristics [21].

In the past, most flue gas dechlorine studies have been conducted under fixed-bed reactor conditions [22,23,24], where gas–solid contact patterns, residence times, and mass transfer processes differ significantly from the dry injection process widely employed in industrial applications. Therefore, previous experimental results cannot be directly applied to guide engineering-scale implementation. In this context, the present study utilizes a laboratory-scale dry injection experimental system to systematically investigate the dechlorination performance of ethanol-digested high-activity calcium-based agents and their effects on mercury transformation and migration under dry injection conditions. Combined with advanced characterization techniques and theoretical simulations, this work aims to elucidate the evolution of active sites, chlorine fixation mechanisms, mercury adsorption, and removal behavior, providing a scientific basis and technical support for the development of high-performance dry flue gas dechlorination and multipollutant control technologies.

## 2. Materials and Methods

### 2.1. Experimental System

**Figure 1:**
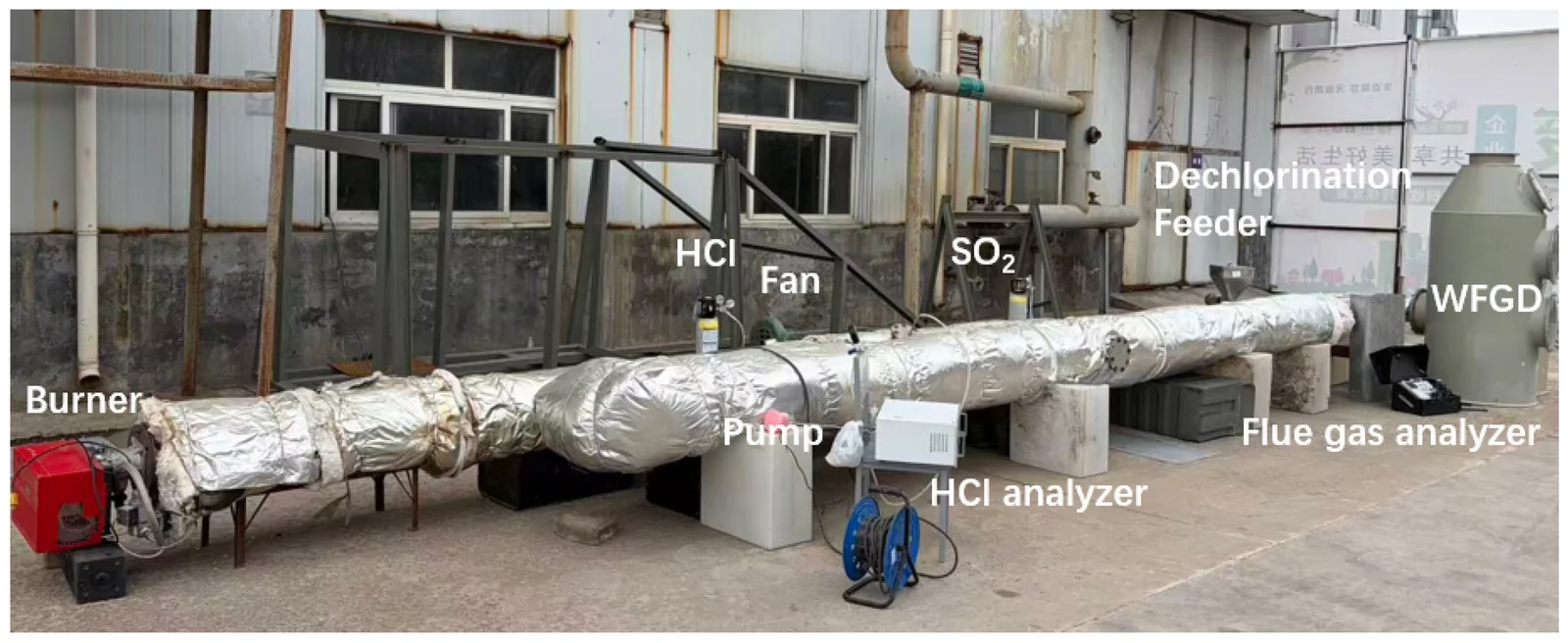
Reaction system of simulated coal-fired flue gas HCl removal.

As shown in Figure 1, the flue gas injection dechlorination experimental system mainly consists of a burner, an injection reactor, and an off-gas treatment unit. A propane burner serves as the heat source to generate high-temperature baseline flue gas, into which hydrogen chloride (HCl) and sulfur dioxide (SO_2_) gases are introduced through mass flow controllers. A blower is used to supply carrier gas to simulate the typical composition of coal-fired flue gas. The total flue gas flow rate is controlled at approximately 600 m^3^/h, with a flue gas velocity of about 5 m/s. The reaction temperature is maintained at 250–300 °C, with HCl and SO_2_ concentrations set at 14 m^3^/h and 800 m^3^/h, respectively. The system is equipped with a fly ash inlet and a dechlorinating agent feeder. The HCl concentration in the flue gas is measured in real time using a laser-based HCl flue gas analyzer (eLAS20, Wuhan Shengnuo Co., Ltd., Wuhan, China), while SO_2_ and O_2_ concentrations are monitored using a flue gas analyzer (J2KN, ECOM GmbH, Iserlohn, Germany). This setup enables dynamic evaluation of the dechlorination process. The treated flue gas is finally purified through an acid-removal unit before being discharged.

### 2.2. Preparation of Dechlorinating Agent

Similar to our previous work [17], firstly, a certain amount of calcium oxide (CaO) was slowly added into an ethanol–water mixed solution (ethanol volume fraction ~33%) to control the digestion reaction rate and particle growth behavior through the ethanol–water system. After completion of the digestion reaction, the resulting suspension was allowed to age for a certain period to further stabilize the particle structure. The solid product was then separated by vacuum filtration and subsequently dried in a thermostatic oven to constant weight, yielding the ethanol-digested calcium-based dechlorinating agent, designated as CaO-E.

To investigate the effect of fly ash doping on dechlorination performance, three fly ash samples from different coal-fired power plants were selected, labeled as FA-1, FA-2, and FA-3. Fly ash-doped samples were prepared by thoroughly mixing ethanol-digested CaO and fly ash at a mass ratio of 1:1. The resulting doped samples were designated as CaO-E/FA-1, CaO-E/FA-2, and CaO-E/FA-3, ensuring sample homogeneity and enabling comparative evaluation of dechlorination performance. For comparison, a calcium-based dechlorinating agent prepared by digestion in pure water was also synthesized and designated as CaO-W.

### 2.3. DFT Calculation

Density functional theory (DFT) simulations were performed using the CP2K 8.2 software to calculate the adsorption energies of gas molecules, including HCl, SO_2_, Hg^0^, and HgCl_2,_ on the surface of the dechlorinating agents [25]. The PBE functional was employed, and the DZVP-MOLOPT-SR-GTH basis set was used. Dispersion interactions were corrected using the DFT-D3 (BJ) scheme [26]. A vacuum layer of 15 Å was applied in the direction perpendicular to the slab model to minimize the interaction between periodic images.

### 2.4. Characterization Device

X-ray diffraction (XRD) patterns were recorded on a D8-ADVANCE diffractometer (Bruker, Billerica, MA, USA). Specific surface area and pore volume were determined by the Brunauer–Emmett–Teller (BET) method at 77 K using an Autosorb-iQ nitrogen adsorption analyzer (Anton Paar, Graz, Austria). The surface morphology of the samples was characterized by scanning electron microscopy (SEM) using a JSM-7401F analyzer (JEOL, Tokyo, Japan) at an accelerating voltage of 15 kV. Fourier transform infrared (FTIR) spectra were acquired with a Nicolet 6700 FTIR spectrophotometer (Thermo Fisher Scientific, Waltham, MA, USA). Particle size distribution in the nanoscale range was measured by dynamic light scattering (DLS) using a Zetasizer Nano ZS 90 (Malvern Instruments, Malvern, UK). Thermogravimetry-mass spectrometry (TG-MS) analysis was performed on a Thermo Plus EVO2/Thermo Mass Photo instrument (Rigaku, Tokyo, Japan).

## 3. Results and Discussions

### 3.1. Physicochemical Characterization

**Figure 2:**
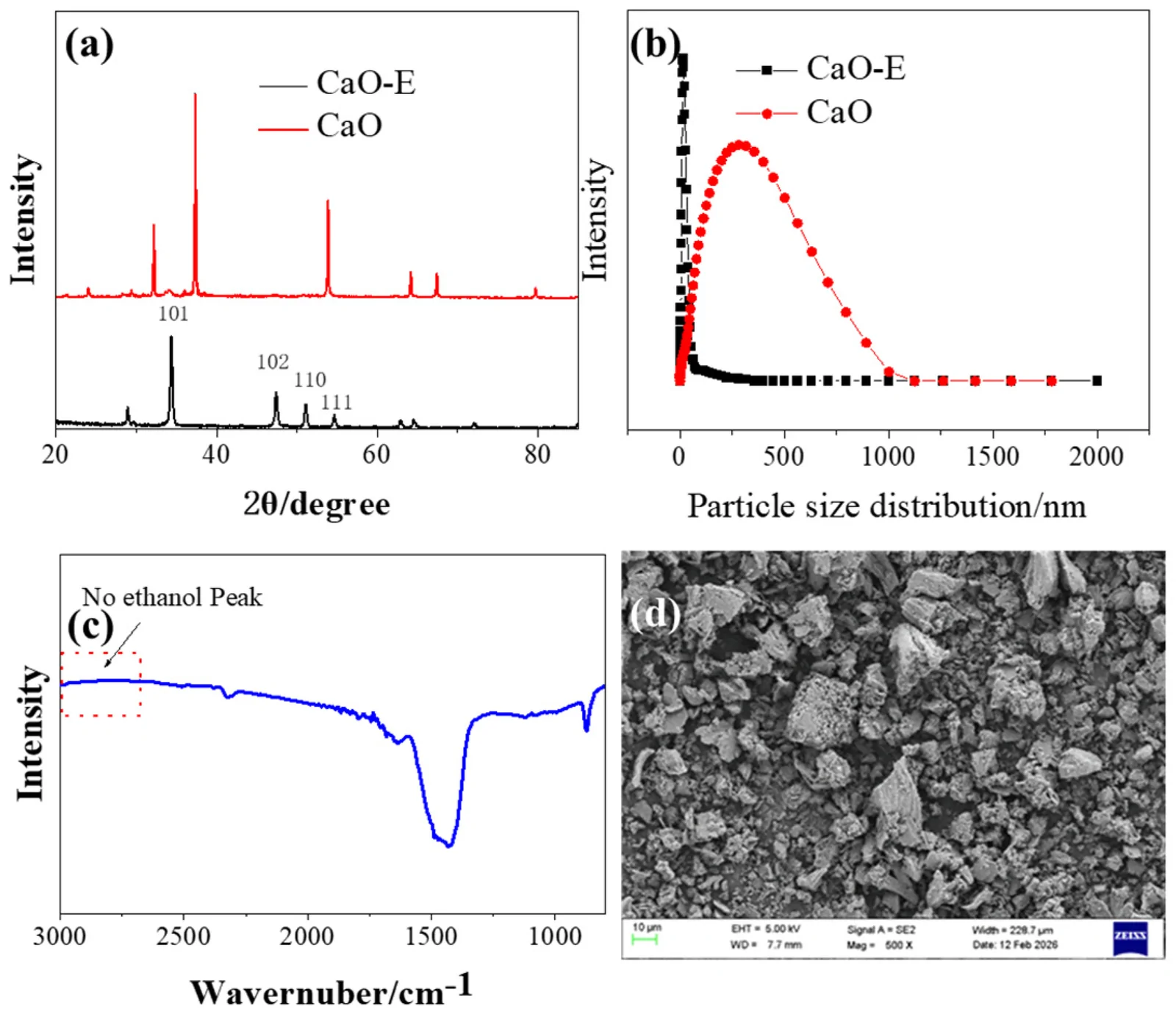
Characterization of the dechlorinating agent. (**a**) X-ray diffraction pattern. (**b**) Laser particle size analysis. (**c**) Fourier transform infrared (FTIR) spectroscopy. (**d**) Scanning electron microscopy (SEM) image.

The physicochemical properties of the dechlorinating agent were systematically analyzed using a variety of characterization techniques. Firstly, XRD (in Figure 2a) was employed to examine the phase composition and crystal structure of the samples. The results indicate that after ethanol digestion, the original calcium oxide (CaO) was completely converted into calcium hydroxide (Ca(OH)_2_), which serves as the primary active component for dechlorination in the system. Notably, the XRD patterns of Ca(OH)_2_ exhibit abundant and complex diffraction peaks corresponding to multiple crystal planes, such as the (101), (102), and (111) planes, suggesting that a diverse set of crystal facets is exposed on the surface. This phenomenon implies that during the CaO digestion process, ethanol molecules significantly inhibit the growth of specific crystal planes, and this effect is generally observed across multiple planes, thereby regulating the crystal morphology of Ca(OH)_2_ and the exposure of active sites.

Subsequently, BET nitrogen adsorption–desorption analysis was performed to investigate the pore structure of ethanol-digested CaO (CaO-E) and water-digested CaO (CaO-W). The results show that the specific surface area of CaO-E is significantly higher than that of CaO-W, measuring 28.32 m^2^/g and 12.94 m^2^/g, respectively. The average pore diameters of CaO-E and CaO-W were 1.1 nm and 1.4 nm, respectively. These results indicate that ethanol digestion effectively suppresses pore collapse while retaining the pore-forming effect of water digestion, promoting the formation of microporous structures. This leads to a more developed porous Ca(OH)_2_ structure, which is favorable for HCl adsorption and reaction. Therefore, ethanol digestion substantially optimizes the specific surface area and pore structure of the dechlorinating agent, providing an important microstructural basis for efficient dechlorination.

Particle size distribution analysis (Figure 2b) indicates that ethanol digestion significantly controls the particle size and distribution characteristics. Compared to the original sample, the digested particles are noticeably smaller and exhibit a more concentrated size distribution. This suggests that ethanol molecules locally hinder the contact between CaO and water during the reaction, suppressing excessive growth of Ca(OH)_2_ crystals and promoting their precipitation in smaller sizes. Previous studies have shown that smaller particle sizes favor enhanced gas–solid reactions. Under actual flue gas conditions, smaller particles can disperse more uniformly, improving gas–solid contact efficiency, accelerating dechlorination kinetics, and enhancing overall dechlorination efficiency.

FTIR in Figure 2c further reveals the functional group characteristics on the surface of the dechlorinating agent. No characteristic vibration peaks of ethanol were observed in the 3000–2700 cm^−1^ region, indicating the absence of residual ethanol on the sample surface and suggesting that ethanol modifies the dechlorinating agent mainly through structural regulation rather than chemical modification. Finally, SEM, shown in Figure 2d, was used to analyze the microstructure of CaO-E. The results show that the sample surface exhibits a typical loose and porous structure. In addition to a small number of dispersed nanoparticles, numerous clusters formed by agglomerated Ca(OH)_2_ particles were observed, most of which exceed 1 μm in size. This morphological feature is highly consistent with ethanol-digested CaO reported in the literature [15]. Comparative analysis further indicates that the samples in this study show good consistency with typical high-performance Ca(OH)_2_ in terms of overall structure and agglomeration behavior, thereby confirming their nanoscale structural characteristics.

**Figure 3:**
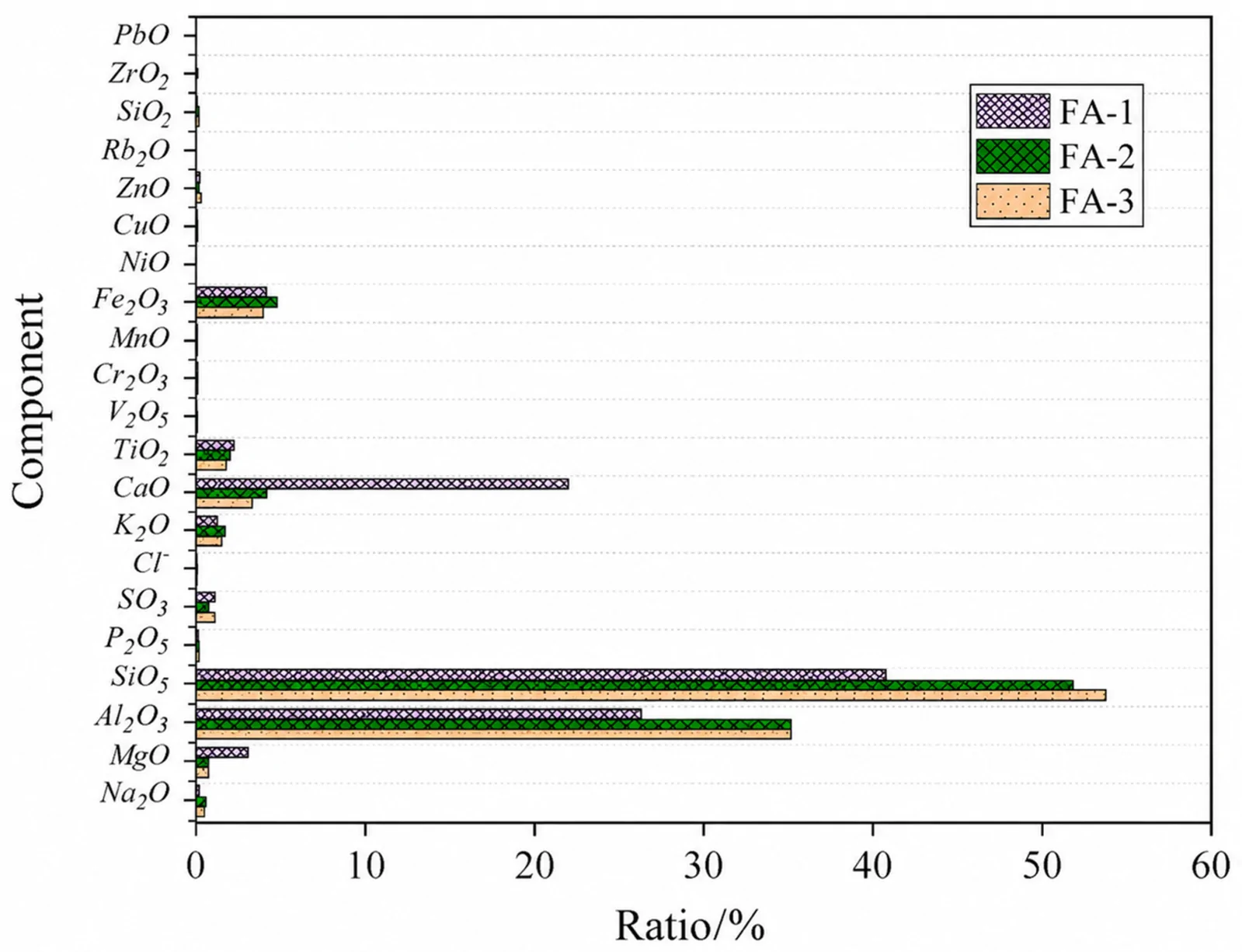
XRF of different fly ashes.

X-ray fluorescence (XRF) characterization was performed on three fly ash samples, as shown in Figure 3. The results indicate that all three samples are primarily composed of Al_2_O_3_ and SiO_2_, with their combined content exceeding 85%, suggesting that the main framework of the fly ash is a typical aluminosilicate structure. Aluminosilicate structures are generally considered relatively inert and contribute little to adsorption [27]. In addition, the fly ash contains a certain amount of CaO, Fe_2_O_3_, K_2_O, TiO_2_, and MgO, as well as various trace heavy metal oxides (e.g., V, Cr, Mn, Ni, Cu, Zn, Pb). Among these, CaO, MgO, and Fe_2_O_3_ are potential deacidification components. Notably, FA-1 contains as much as 22.3% CaO, which may confer potential for its use as a calcium-based dry dechlorinating agent [28].

### 3.2. Dechlorination Performance

**Figure 4:**
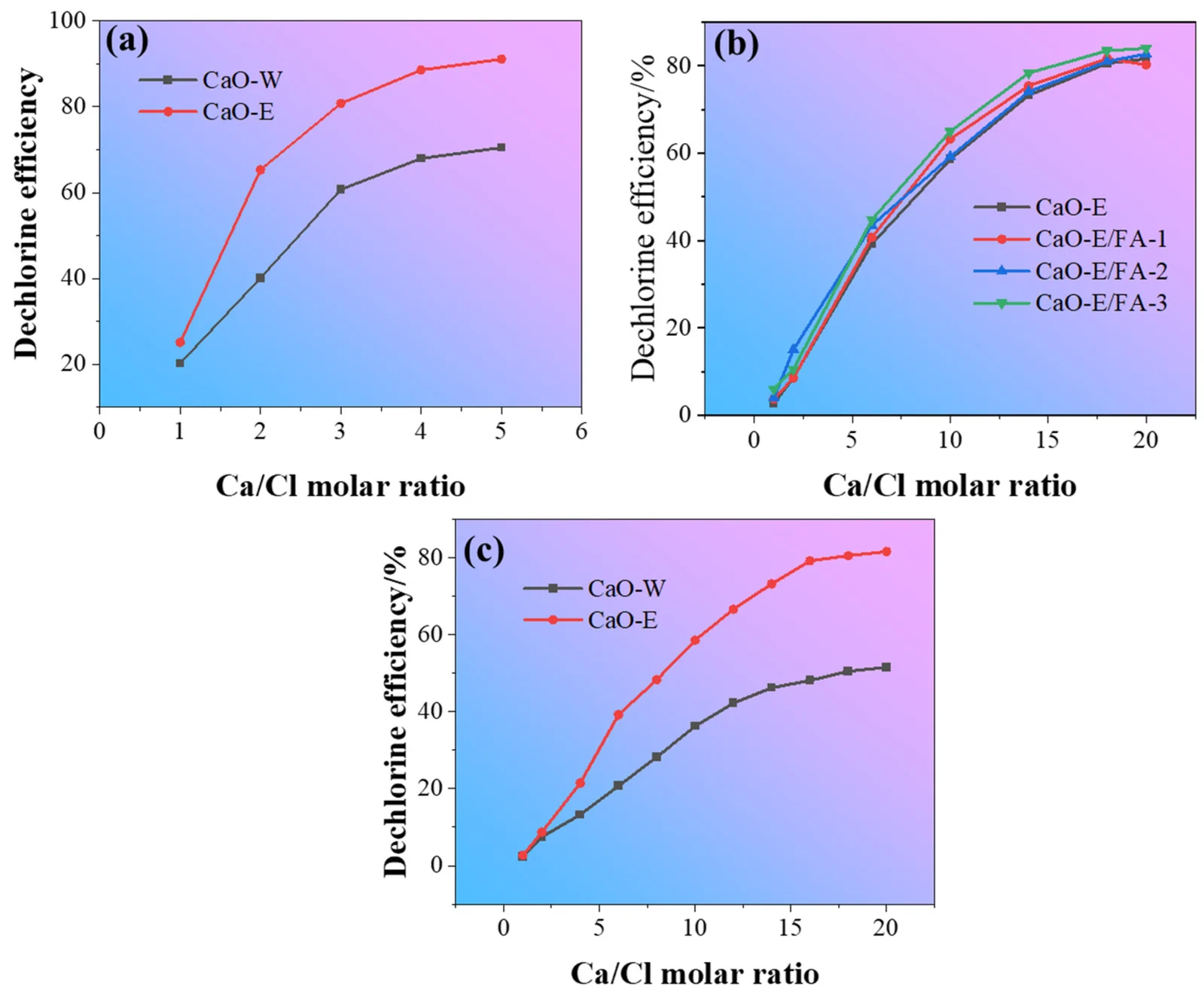
Experimental results of spray dichlorination. (**a**) Relationship between dechlorination efficiency and calcium-to-chlorine molar ratio. (**b**) Effect of fly ash on dechlorination efficiency. (**c**) Effect of sulfur dioxide on dechlorination efficiency.

As shown in Figure 4a, under conditions without SO_2_ introduction, CaO-E exhibits significantly superior dechlorination performance compared to CaO-W. When the Ca/Cl molar ratio, which is defined as the molar amount ratio of injected Ca(OH)_2_ and HCl in flue gas, is increased to 5, the dechlorination efficiency stabilizes at approximately 90%. This result indicates that ethanol-assisted digestion effectively improves the physicochemical properties of the adsorbent, allowing the active component to interact more fully with HCl during the reaction. Generally, ethanol digestion helps form a looser structure with a larger specific surface area; simultaneously, the resulting porous structure facilitates gas diffusion into the particle interior, thereby enhancing the utilization efficiency of active sites. Consequently, under the same Ca/Cl ratio, CaO-E demonstrates higher conversion capacity.

Further analysis reveals that when the Ca/Cl ratio is increased from 4 to 5, the improvement in dechlorination efficiency becomes less pronounced, indicating that the system in this range is approaching a mass transfer- or reaction equilibrium-controlled stage, and newly added active sites are not fully utilized. The generated products may cover part of the active sites, thus limiting subsequent reactions [29]. Therefore, simply increasing the Ca/Cl ratio does not lead to a proportional gain in efficiency. So, the optimal reaction conditions of Ca/Cl ratio were ~4.0–5.0, as the dechlorination efficiency can be ensured with a smaller amount of adsorbent.

Figure 4b shows that the addition of fly ash has a relatively limited effect on overall flue gas dechlorination. Under the same operating conditions, the introduction of fly ash only slightly improves HCl removal efficiency, and the overall reaction trend is basically consistent with that observed without fly ash, suggesting that fly ash is not the dominant factor controlling dechlorination performance. This minor promotional effect may be attributed to the additional surface area provided by the fly ash and the small amounts of alkaline components it contains, which can offer auxiliary sites for HCl adsorption or intermediate reactions and improve the dispersion of the dechlorinating agent in the flue. Overall, fly ash mainly plays a supporting or synergistic role and cannot fundamentally alter the controlling steps of the dechlorination process.

### 3.3. Effect of Sulfur Dioxide on Dechlorination

For coal-fired flue gas, SO_2_ is a very important acidic gas, and its concentration is usually higher than that of HCl. Currently, most coal-fired power plants use wet flue gas desulfurization (WFGD) systems, which inevitably causes the flue gas dechlorination process to be affected by SO_2_. As shown in Figure 4c, after the introduction of SO_2_, the dechlorination efficiency changes significantly. Compared with SO_2_-free conditions, the HCl removal efficiency of CaO-E decreases markedly. Even when the Ca/Cl ratio is increased to 15, the dechlorination efficiency can only be maintained at approximately 80%, indicating that a sulfur-containing atmosphere has a pronounced negative effect on HCl removal.

The primary reason for this phenomenon is the surface coverage and pore blockage caused by sulfate species formed during the reaction [30]. The products generated from the reaction between SO_2_ and the active components tend to deposit on the outer layer of the particles, gradually forming a dense product layer. This layer hinders the diffusion of HCl into the particle interior, making it difficult for a large portion of potential reactive calcium sources to participate further in the reaction, thereby significantly reducing the effective utilization of calcium [31]. As the reaction proceeds, the system is more likely to shift from intrinsic reaction control to diffusion-limited control; therefore, the efficiency improvement achieved by simply increasing the Ca/Cl ratio becomes limited.

In addition, competitive reactions between sulfur- and chlorine-containing species may further weaken the dechlorination process. The consumption of SO_2_ occupies part of the active sites and alters the chemical environment of the particle surface [32], reducing the number of effective sites available for chlorination reactions. This synergistic inhibitory effect collectively leads to a decrease in overall dechlorination efficiency. Despite these adverse effects, CaO-E still performs better than CaO-W. Under identical dosing conditions, the ethanol-digested sample continues to exhibit higher removal efficiency, indicating that its modified structural features partially alleviate the mass transfer limitations caused by product layer formation. The more developed pore structure and higher accessibility facilitate gas access to reactive regions not yet blocked by sulfates and delay particle deactivation.

**Figure 5:**
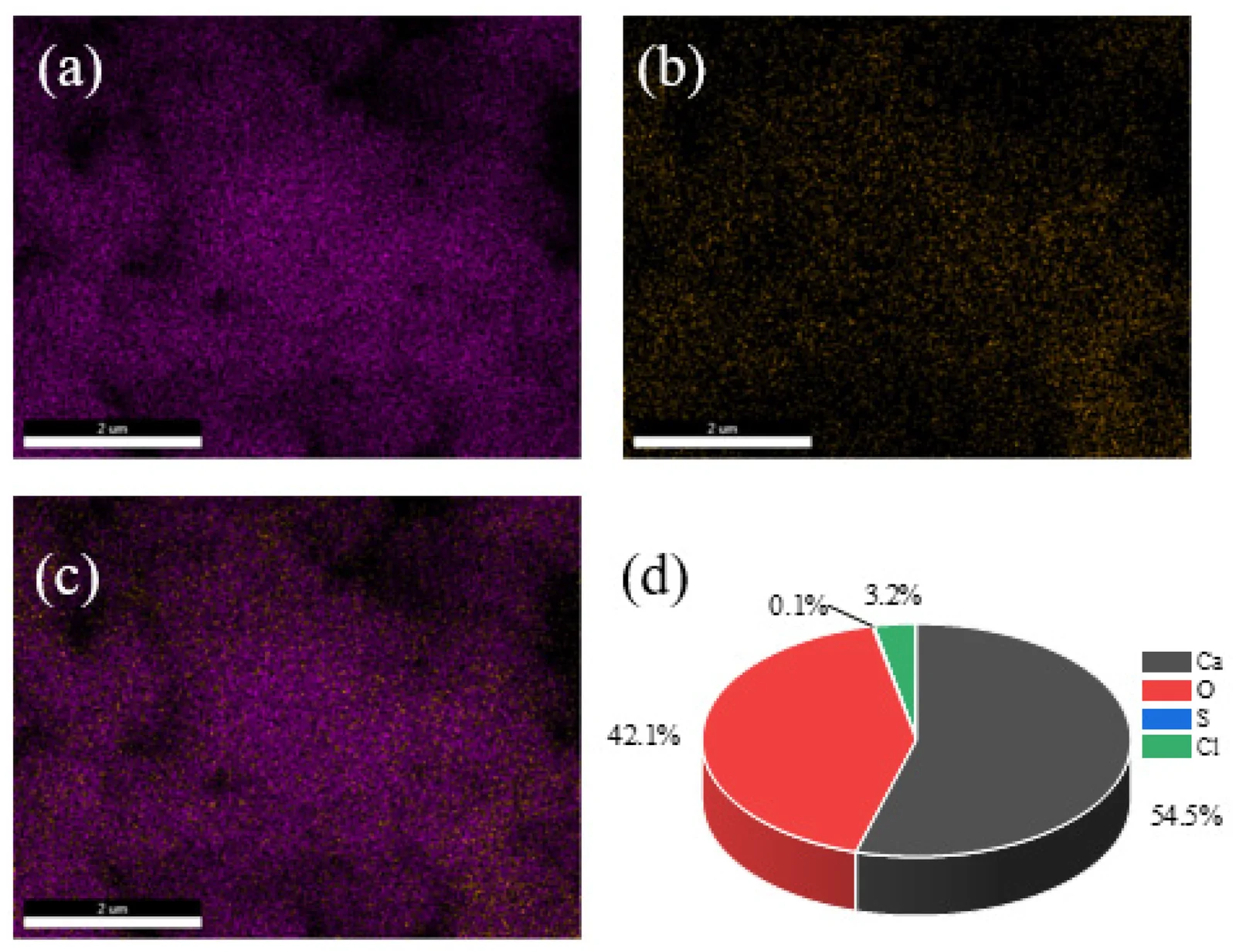
Transmission electron microscopy combined with energy dispersive spectroscopy: (**a**) Chlorine element distribution on the surface of the dechlorinating agent. (**b**) Sulfur element distribution on the surface of the dechlorinating agent. (**c**) Combined distribution of chlorine and sulfur on the surface of the dechlorinating agent. (**d**) Surface element proportion of the dechlorinating agent measured by energy dispersive spectroscopy.

To further elucidate the inhibitory mechanism of SO_2_ on the dechlorination reaction, EDS elemental analysis was performed on the samples after the injection dechlorination experiments to characterize the distribution and relative contents of sulfur and chlorine on the surface (Cl 1.34 at.%, S 0.05 at.%), as shown in Figure 5. The red regions represent chlorine distribution, while the yellow regions represent sulfur distribution. The results show that chlorine is significantly more enriched on the sample surface than sulfur, indicating that under these conditions, the adsorption and reaction of HCl on the particle surface dominate, while the competitive adsorption of SO_2_ is relatively weak. This is consistent with our previous works in fixed-bed experiments, where results indicated that the dominant inhibitory effect of SO_2_ arises from the blockage of active sites, while competitive adsorption plays a secondary role [15,16]. Therefore, during the flue gas dechlorination process, the reactivity of HCl is significantly higher than that of SO_2_.

### 3.4. Reaction Path Analysis

**Figure 6:**
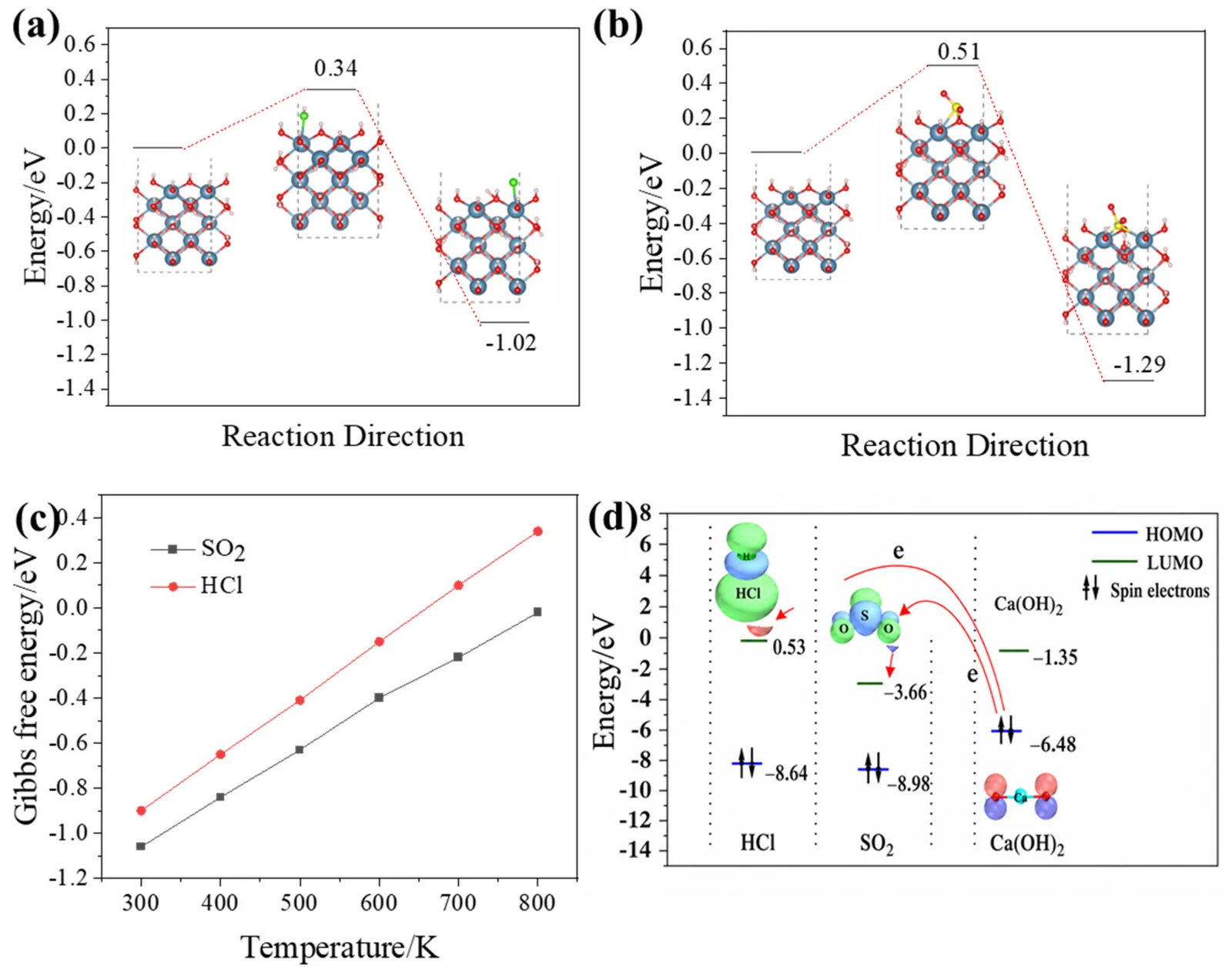
(**a**) Reaction pathway of dechlorination reaction. (**b**) Reaction pathway of desulfurization reaction. (**c**) Free energy changes of dechlorination and desulfurization. (**d**) Frontier orbital distributions of HCl and SO_2_.

The dechlorination reaction is generally considered to proceed in two steps, with the first step—the formation of the CaClOH intermediate—playing the dominant role. In this study, based on density functional theory (DFT), the elementary reaction pathway for the formation of the CaClOH intermediate at calcium sites on the dechlorinating agent surface was calculated, exhibited in Figure 6a. The results show that the reaction barrier is approximately 0.34 eV. This relatively low energy barrier provides favorable conditions for achieving high dechlorination efficiency.

Reaction energy calculations indicate that the total energy of the dechlorinating agent decreases by 1.02 eV after the dechlorination reaction, suggesting that a large reaction energy release favors the stable existence of dechlorination products. In addition, the elementary reaction pathway for SO_2_ removal was also calculated, which was present in Figure 6b. The results show that the energy barrier for the desulfurization reaction is 0.51 eV, which is higher than that of the dechlorination reaction, indicating that the dechlorination process exhibits stronger selectivity compared to desulfurization. The total energy decrease of the desulfurization elementary reaction is −1.29 eV, suggesting that the desulfurization products possess higher thermodynamic stability. The changes in reaction Gibbs free energy for both dechlorination and desulfurization processes in the temperature range of 300–800 K further support this conclusion, as shown in Figure 6c.

Furthermore, the adsorption energy of HCl under co-adsorption conditions with SO_2_ was calculated. The adsorption energy of HCl in this case is −1.2 eV, indicating a slight reduction in adsorption strength at adjacent sites, which may be attributed to the weakened coordination ability of calcium sites induced by SO_2_. In addition, analysis of molecular frontier orbitals reveals that the lowest unoccupied molecular orbital (LUMO) energy level of SO_2_ (−3.66 eV) is lower than that of HCl (0.53 eV), making SO_2_ more prone to electron acceptance from the dechlorinating agent, thereby leading to more stable final products. Therefore, the low frontier orbital energy level of SO_2_ is an important reason for its inhibitory effect on dechlorination efficiency.

**Figure 7:**
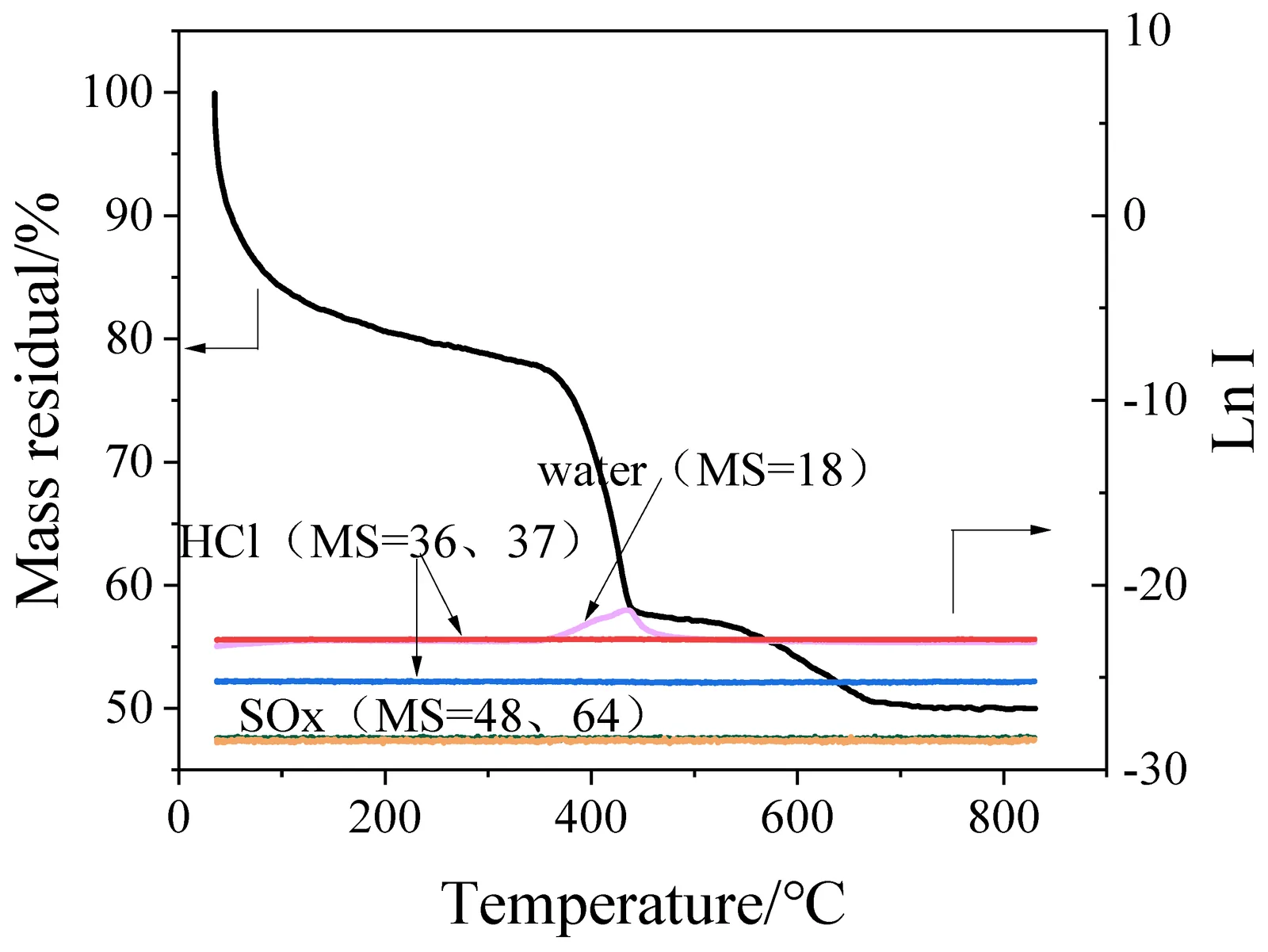
Characterization of dechlorination products by TG-MS. (Black line means the mass residual, and other color lines mean signal intensity of LnI).

To further investigate the reaction products, thermogravimetric–mass spectrometry (TG–MS) analysis was conducted, as shown in Figure 7. The temperature was ramped from room temperature to 800 °C, and the mass spectrometer monitored the signals of HCl, SO_2_, H_2_O, and SO species during heating. The results show a strong signal corresponding to the release of crystalline water during heating, while the signals of HCl and SO*_x_* are relatively weak. This indicates that the formed chlorides and sulfides exist in strongly chemically bound and relatively stable forms. Among them, the SO*_x_* signal is the weakest, suggesting that calcium sulfate products are more stable than calcium chloride products, which is consistent with the DFT results discussed above. Therefore, it can be concluded that flue gas dechlorination effectively immobilizes pollutants in stable forms that are difficult to desorb, thereby minimizing the risk of secondary pollution.

### 3.5. On Mercury Speciation and Concentration

**Figure 8:**
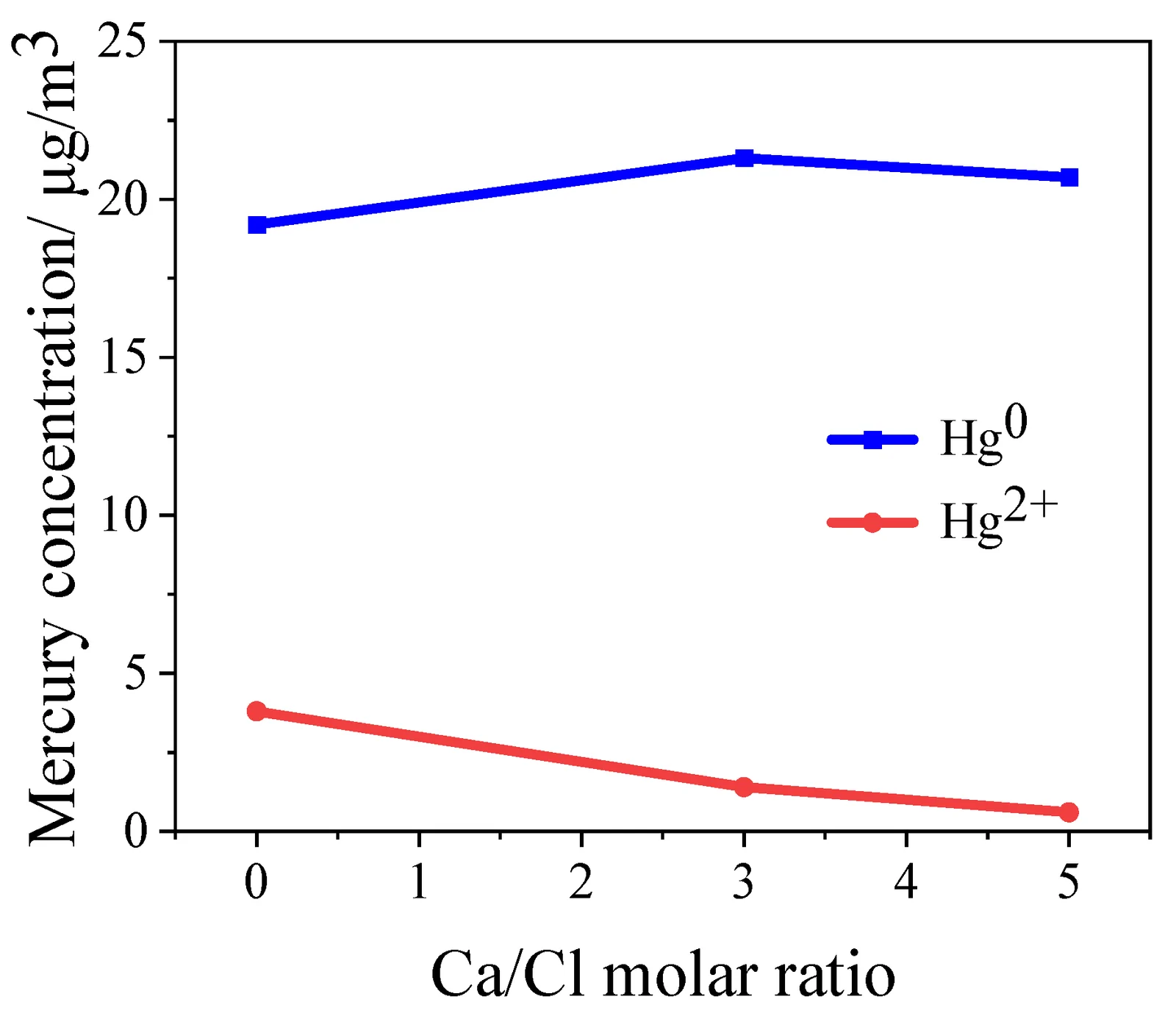
Effect of flue gas dechlorination on mercury speciation and concentration.

During the experiment, a stream of mercury-containing flue gas was introduced into the reactor. Before the injection of ethanol-digested calcium oxide, the concentrations of elemental mercury (Hg^0^) and divalent mercury (Hg^2+^) in the blank flue gas were 19.2 μg/m^3^ and 3.8 μg/m^3^, respectively. After injecting ethanol-digested CaO, at Ca/Cl molar ratios of 3 and 5, the Hg^0^ concentrations in the outlet flue gas were 21.3 μg/m^3^ and 20.7 μg/m^3^, showing little change compared with the blank conditions. In contrast, the Hg^2+^ concentrations decreased significantly to 1.4 μg/m^3^ and 0.6 μg/m^3^, respectively. These results in Figure 8 indicate that ethanol-digested CaO has a limited removal effect on Hg^0^ but can effectively reduce Hg^2+^ concentrations in flue gas.

The main reason is that Hg^0^ is chemically stable and highly volatile, interacting weakly with the adsorbent surface, making it difficult to capture directly [33]. Hg^2+^, on the other hand, mainly exists as chlorine-containing species such as HgCl_2_, which are highly polar and acidic and can readily react with the basic active sites on the surface of ethanol-digested CaO, leading to adsorption and removal [34]. Additionally, during flue gas dechlorination, as the Ca/Cl ratio increases, the concentration of chlorine-containing species in the flue gas gradually decreases. Given that the oxidation of Hg^0^ to Hg^2+^ primarily occurs through the reaction Hg^0^ + HCl + O_2_, it would reduce the oxidation rate of Hg^0^ to Hg^2+^ and thereby inhibit Hg^2+^ formation at the source. Under the combined effect of reduced chlorine availability and adsorption at basic sites, the Hg^2+^ concentration in flue gas decreases further with increasing Ca/Cl ratio.

**Figure 9:**
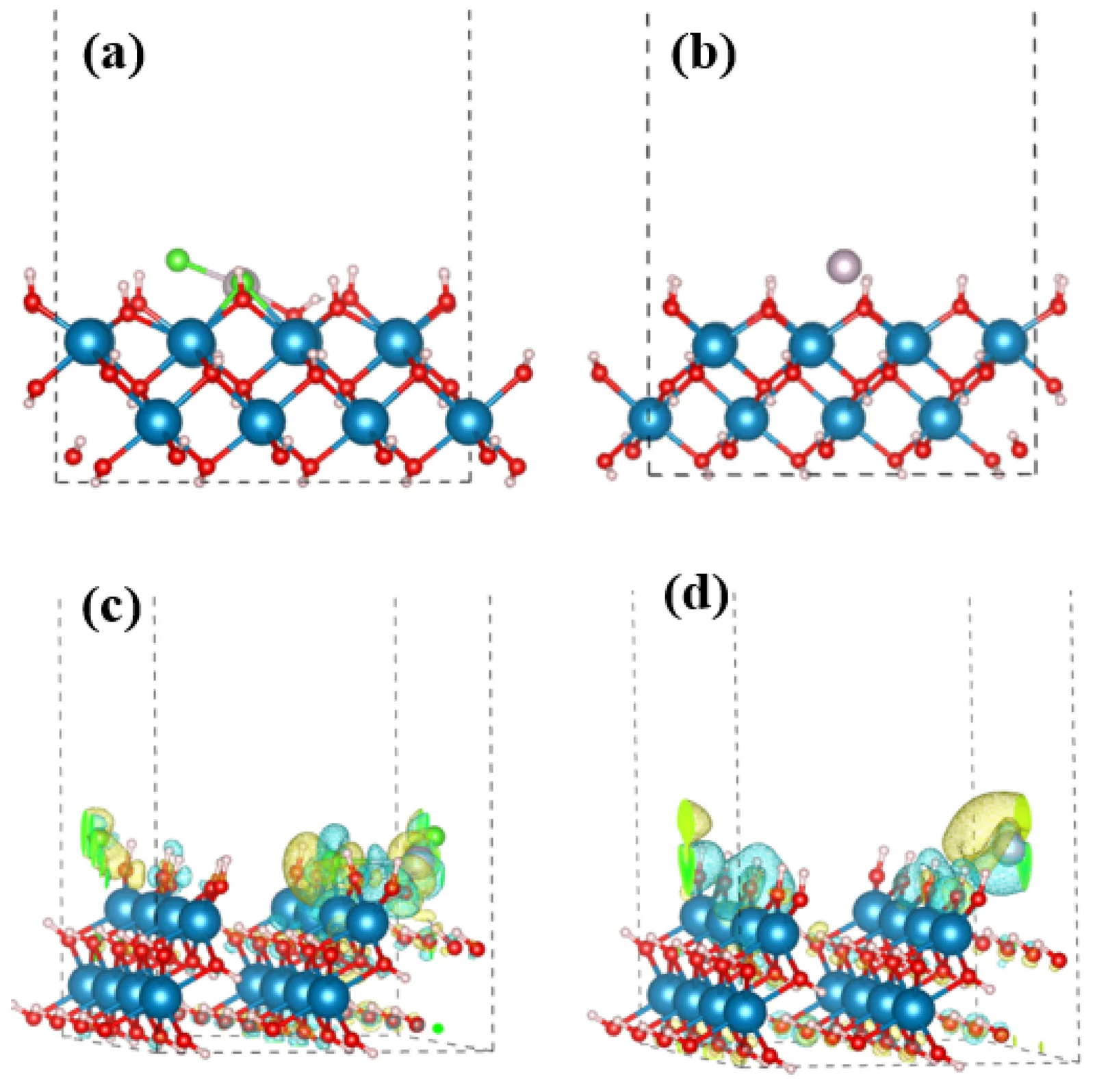
Adsorption configurations and EDD maps of HgCl_2_ and Hg^0^. (**a**) Adsorption configuration of HgCl_2_. (**b**) Adsorption configuration of Hg^0^. (**c**) EDD map of HgCl_2_ adsorption. (**d**) EDD map of Hg^0^ adsorption in ±0.001 e/Bohr^3^ isosurface (Yellow: electron accumulation; cyan: electron depletion).

Density functional theory (DFT) was employed to investigate the adsorption capacity of the dechlorinating agent toward Hg^0^ and HgCl_2_, in order to elucidate the mechanism by which flue gas dechlorination affects mercury speciation and concentration. First, the adsorption configurations were geometrically optimized. Based on the optimized adsorption structures on the surface of the dechlorinating agent of Figure 9a,b, a significant difference in adsorption behavior between Hg^0^ and HgCl_2_ can be observed. The calculated adsorption energies are −0.11 eV and −0.57 eV, respectively, indicating that Hg^0^ is mainly physically adsorbed on the surface of the dechlorinating agent through weak van der Waals interactions [35], whereas HgCl_2_ exhibits typical chemisorption characteristics. This difference can be attributed to the stable outer electron configuration and strong chemical inertness of Hg^0^, which make it difficult to form strong chemical bonds with the surface of the dechlorinating agent. In contrast, HgCl_2_, due to multi-body interactions and the Lewis acidity of Hg^2+^, can form stable bonds with surface oxygen and calcium species. In the electron density difference (EDD) maps of Figure 9c,d, obvious electron accumulation is observed between the Cl atoms of HgCl_2_ and Ca, as well as between Hg and O, further confirming that chemical bonds are formed between HgCl_2_ and the dechlorinating agent surface, leading to its strong adsorption capability [21]. Therefore, during flue gas dechlorination, the dechlorinating agent has almost no effective capture ability for elemental mercury, while it can achieve efficient and stable removal of oxidized mercury species.

### 3.6. Economic Analysis

Taking a 300 MW unit as an example, an economic assessment of flue gas dechlorination was conducted. According to data obtained from the DCS system under full-load operating conditions, the standard-state flue gas flow is approximately 1.2 × 10^6^ Nm^3^/h, the coal feed rate is 135 t/h, and the SO_2_ concentration is around 800 mg/Nm^3^. Elemental analysis of the coal indicates that the chlorine content entering the furnace is approximately 138.39 mg/kg. Assuming a chlorine volatilization rate of 90%, the predicted HCl concentration in the flue gas is approximately 14 mg/Nm^3^. Further, assuming that 4% of HCl is converted to gypsum-bound chlorine, the estimated chlorine content in the gypsum is about 290 mg/kg.

Based on test data from a small-scale high-temperature experimental platform, the economic feasibility of implementing flue gas injection dechlorination for the 300 MW unit was further evaluated. Target control objectives included wastewater reduction and 60%, 70%, and 80% reductions in chlorine content of the desulfurization gypsum, and system operating costs under different scenarios were compared. In the cost accounting process, practical industrial conditions were fully considered. The price of calcium oxide was based on commonly available 85% purity industrial-grade material; during the calculation of the Ca/Cl ratio, the content of the effective component was corrected accordingly. The inlet HCl concentration in the flue gas was taken as 14 mg/Nm^3^, and the corresponding Ca/Cl ratio was adjusted according to the efficiency conversion described previously.

**Figure 10:**
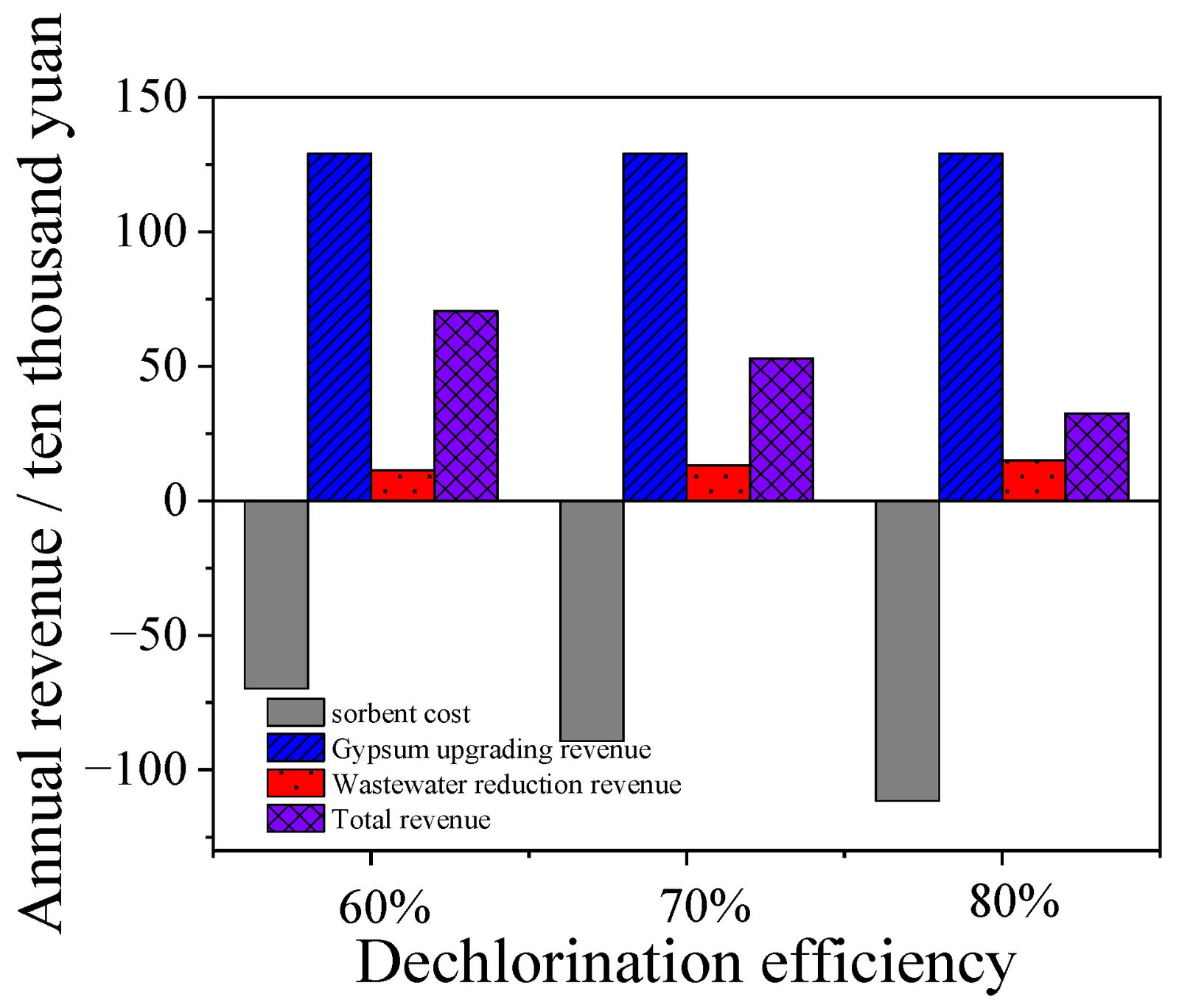
Economic analysis of flue gas dechlorination.

Under current market conditions, when the chlorine content in gypsum is below 100 mg/kg, the value of high-quality gypsum increases by approximately 100 CNY/t. Therefore, the economic benefit from gypsum upgrading was calculated as the product of gypsum production and the unit value increase. Conventional desulfurization wastewater treatment costs were included at 40 CNY/t. Ethanol solution recovery was assumed, so its cost was not included. Annual costs and revenues were calculated based on 5000 operating hours. The comprehensive economic benefit of flue gas dechlorination was defined as the sum of the wastewater reduction benefit and the gypsum upgrading benefit minus the cost of the dechlorinating agent. The calculated dechlorination efficiencies under different scenarios are shown in Figure 10 below.

From the perspective of the economics of flue gas dechlorination, the economic benefit of gypsum upgrading is far greater than that of wastewater reduction. As the dechlorination efficiency of flue gas increases, the cost of the dechlorinating agent gradually rises, while the benefit from gypsum upgrading remains constant and the benefit from wastewater reduction increases only slightly. This leads to a noticeable decline in total net benefit. From an economic perspective, this indicates that flue gas dechlorination achieves optimal economic benefit within a low-to-moderate efficiency range, whereas pursuing extremely high dechlorination efficiencies results in significant diminishing marginal returns. When the dechlorination efficiency exceeds a certain level, the rate of increase in reagent cost far outpaces the growth of benefits from wastewater reduction and gypsum upgrading, making further improvements in dechlorination efficiency contribute little to total economic gain. Therefore, in industrial applications, it is crucial to reasonably control the dechlorination efficiency within the optimal benefit range to maximize economic returns. In other words, overemphasizing a high dechlorination rate not only increases operating costs but may also extend the investment payback period, making it economically and technically suboptimal. Moreover, it is important to note that an effective strategy for handling the spent dechlorinating agent involves recovering it together with fly ash and subjecting the mixture to mechanochemical modification. This treatment breaks down the product layer covering the adsorbent surface and exposes fresh active sites, thus enhancing the overall cost-effectiveness of flue gas dechlorination. Nevertheless, the potential deterioration in fly ash quality due to elevated chloride levels must be carefully evaluated.

## 4. Conclusions

Based on the experimental and theoretical investigations, the following conclusions can be drawn:

(1) The CaO-based dechlorinating agent prepared via ethanol digestion (CaO-E) exhibits notably higher dechlorination efficiency than that prepared with pure water (CaO-W).
(2) The addition of fly ash marginally enhances the dechlorination performance, whereas the presence of SO_2_ severely suppresses the reaction.
(3) Density functional theory (DFT) calculations reveal that SO_2_ possesses low-lying frontier orbital energies, which facilitate stronger binding to basic sites and yield more stable products, thus accounting for its inhibitory effect.
(4) Despite the SO_2_ inhibition, the dechlorination reaction is kinetically favored over desulfurization, thereby ensuring its preferential occurrence under the tested conditions.
(5) The dechlorination process exerts negligible influence on elemental mercury (Hg^0^) but significantly promotes the removal of oxidized mercury (HgCl_2_).

## Data Availability

The original contributions presented in this study are included in the article. Further inquiries can be directed to the corresponding author.
